# Ethics and non-evidence based therapies: Portuguese perspective in a global setting

**DOI:** 10.1007/s40592-022-00172-6

**Published:** 2022-12-31

**Authors:** João Madruga Dias

**Affiliations:** 1 Rheumatology Department, Unidade de Torres Novas, Centro Hospitalar do Médio Tejo EPE, R. Xanana Gusmão 45, 2350-754 Torres Novas, Portugal; 2 Faculdade de Ciências Médicas, Universidade Nova de Lisboa (NOVA Medical School), Campo Mártires da Pátria 130, 1169-056 Lisbon, Portugal

**Keywords:** Clinical ethics, Malpractice, Professional misconduct, Public health ethics, Regulation

## Abstract

A contemporary serious lack of scientific knowledge by the general public and many decision-makers is now quite perceptible, both globally and in Portugal. Living in a science-driven technological world filled with scientific illiteracy is dangerous and a path toward disaster. Recent years brought a fairly strong global movement promoting the so-called “alternative therapy” that also affected Portugal. I propose an evidence-based ethics reflection and argumentation, both encompassing the global and the specific Portuguese reality. I debate the specific arguments used in favour of alternative therapies, demonstrating the inherent fallacies of thought, deliberate manipulation of words and concepts, and the dire consequences for global and local health politics by following this line of biased reasoning.

## Introduction

In current times there is a serious lack of scientific knowledge by the general public and many decision-makers, both globally and in Portugal. The public is bombarded with a spectre that goes from fraud to infotainment and a confusing mixture of quasi-scientific studies whose purpose is no different from click-bait ads. Real scientific breakthroughs are levelled in traditional and social media as being as important as these click-baits (Dias 2017). At the decision-making level economic interests are confused for science, while other players deliberately use confusing scientific jargon to create pseudoscience to suit a political need. As argued by Carl Sagan, living in a science-driven technological world filled with scientific illiteracy is dangerous and a path toward disaster (Sagan 1990).

Recent years brought a fairly strong global movement promoting the so-called “alternative therapy” that also affected Portugal (Carvalho et al. 2012). A possible definition of an alternative therapy incorporates two key elements: its efficacy is either unproven or disproved; and the rationale for testing it in a trial cannot be expressed in acceptable scientific language. (Smith 2016) When robust evidence demonstrates the efficacy of a therapeutic modality, whose underlying mechanisms of action do not violate fundamental scientific and logical principles, that therapy becomes a candidate to be used in medicine and can no longer be considered as an alternative therapy (Smith 2016). Using a now popular adage, alternative medicine that works is simply called medicine (Gavura 2013).

## Materials and methods

The author proceeded to a literature review using PubMed with the terms “ethics” and “alternative medicine” OR “alternative therapy”. Articles published from 2000 until July 2020 were selected in order to sustain the arguments in discussion. The deontological code of the Portuguese medical association and Portuguese legal publications on healthcare were consulted. Healthcare-related publications by international authorities were consulted and quoted when necessary.

## Results and discussion

Alternative therapy is not associated with one specific social or political party or the current or past governments but it is a trend that has been growing in strength and influence across society, motivated by alternative practice interests. These interests include endorsing alternative medicine practitioners and recognizing them as valid health practitioners, approval of alternative “treatments” to be used on patients (without scientific testing), and the perennially present economic backstage. Lobbying and political contacts not only allowed alternative medicine practitioners’ recognition by the Portuguese Health Ministry and other health authorities (Portaria n.º 172-B,C,D,E,F/2015; Portaria nº 45/2018), but even tax exemptions similar to doctors and other health practitioners. Patients can deduct both appointments in their own taxes as if no differences exist in principle, science, training, requirements, and outcomes.

Let’s consider a specific area of medical knowledge: Rheumatology. Up until nearly fifty years ago, the word *rheumatism* defined what are now classified as hundreds of different rheumatic diseases and conditions. Although rheumatologists have state-of-the-art treatments with immunotherapy and expensive biotechnological drugs, the truth is the etiopathology of most conditions still eludes us. Doctors and scientists (and many are both) don’t know everything, nor do they claim to, but what is known is due to science. In recent years, science has allowed more accurate diagnoses, better follow-up, and was able to offer life-changing treatments to patients. These were never available before and the results would have been considered miracles centuries (if not to say decades) ago. Older patients with longstanding destructive disease can still benefit from these new medications, a human time-bridge between old and new Rheumatology: and they attest to the enormous advance provided by science. But surprisingly the claims of alternative practitioners regarding their methods and usage for rheumatic diseases (even inflammatory ones) are as prominent as the ones science provides, minus the evidence. Considering that some claim these alternative practices are millennia old, no doubt accessible by at the very least, the wealthy unhealthy of that time, where are the results?

Some alternative therapy practitioners fail to understand that science is not a belief or faith, it’s a system of proof. More than a body of knowledge, it’s a way of (critical and sceptical) thinking. If science proves a hypothesis wrong, a scientist is compelled to change it. The commonplace fantasy of a common substance, mysterious unknown energy source, or even some exotic unheard-of plant being the cure to some of the most unfortunate diseases that affect humankind is shared by many doctors and alternative practitioners alike. The first realised results have to be proven, and for that the scientific method must be used. But where do you start when the principles behind your practice are inherently wrong (the simplest case being homeopathy (Ernst 2010; House of Commons Science and Technology Committee Evidence Check 2: Homeopathy. 2016), while at the same time you are being validated by a health authority? Shouldn’t health authorities be entirely science-driven?

Some key arguments in favour of alternative therapies will now be explored and rebutted:

*The perspective of consumer autonomy and the free market.* The underlying fallacy of treating healthcare as a consumer product and not as a universal human right, leads to the logical conclusion that consumer autonomy is paramount. If there are unmet needs (orphan and rare diseases, diseases with few or scarcely effective treatments, or other needs not addressed by current scientific therapies), there is a product to meet them, and hence, a consumer for that product. Free market would then dictate the choice of therapeutic regimens. Yet, science does not validate facts by popularity nor is it bound by marketing rules. Lymphoma or rheumatoid arthritis will progress, no matter how well marketed an ineffective product is. In most cases, patients do not have the knowledge or scientific critical thinking tools to understand the fallacies and logical discrepancies behind alternative therapies, and the consequences from delayed or no effective treatment. Moreover, if medicine is considered a commodity, the consumer expects to receive a product with the desired effect. A washing machine should wash, a television should show images. This does not happen with alternative therapies, as successive unbiased randomized clinical trials (when applicable) and systematic reviews of literature have shown (Cochrane Reviews related to Complementary Medicine). It is not ethically or morally acceptable to sell broken products to an unwary customer: an ineffective therapy is a broken product.

*Progress has a need for competition*. Again related to free market perspectives and healthcare as a commodity, this argument stands on (very) thin ice. If alternative therapies are competition, they will drive the remaining healthcare providers to progress and search for better solutions. Yet, while competition drives prices toward the point at which the social allocation of resources is most efficient (Macdonald and Gavura 2016), it can also provide a race to the bottom. Alternative therapies have at best the same results as a placebo. It is very expensive to produce new medications and pharmaceutical companies might just compete with better looking placebos with more robust clinical trials better demonstrating their placebo effect. Considering homeopathy, would it be easier to develop a novel anti-interleukin immunoglobulin that works in specific studied receptors in psoriatic arthritis or a solution consisting of dihydrogen monoxide? Would it be easier to deal with potential severe and unknown side-effects going through phase I till phase IV clinical trials and post-market surveillance, or to have no side-effects? The race to the bottom is actually a race to the top from an economic perspective: water is much cheaper by the litre than solutions of immunoglobulins and there are no research and development costs, as all you need is clever marketing. Another argument against this “competition” is that consumers might not be sufficiently well-informed to make good purchasing decisions (Macdonald and Gavura 2016). Would you buy any homeopathic solution knowing it contains zero active molecules and that the proposed mechanism is not only scientifically obsolete but goes against elementary chemistry and physics? It is ethically inadmissible to pretend that alternative therapies are competition to scientifically proven therapies. Most scientifically proven therapies are state-paid or insurance-covered and assuming alternative therapies as a true competitor, states or private companies could risk wasting valuable resources on ineffective unproven therapies. Moreover, that situation would be promoting a race-to-the-bottom, assuming customers (patients) would be happy with a placebo level of effectiveness.

*The unknown science argument*. According to this argument, alternative therapies may have an effect due to unknown physical properties or forces in action, and hence may reveal new pathways of effective treatment for pathologies. Of course*, primum non nocere* (Sokol 2013), as embedded as it is into modern medical practice, is the counter argument that first comes to mind. An effect has to be proven in order for a treatment to be acceptable for clinical use. But for an effect to be tested, scientists from different scientific areas that interact with medical knowledge (physics, chemistry, biology, etc.) need to measure it and yet they did not encounter any of the unknown physical properties or forces claimed by alternative therapies. Some alternative practitioners state that these alternative therapies have millennia of usage—nevertheless their results were never formally proven. The increased life expectancy and quality of life of modern day humans is due to technological and scientific breakthroughs, many of which applied to medicine: from universal vaccination to hand washing, better and more available food to effective drugs. Humankind had none of these before, but some of their alternative practices existed, without any measurable effect on mortality, morbidity, malnutrition or life expectancy. It seems that if any unknown science is to be found, it is either completely ineffective or it is wrongly applied by these alternative therapies.

*The complementary treatment argument.* Following up on the unknown science argument, the complementary treatment argument claims that even if alternative therapies have no measurable effect besides placebo, no harm is done and patients have nothing to lose. In reality many alternative therapies have side effects, some quite severe and even life-threatening. Two examples include chiropractic manipulation to the immature spine as a serious risk of physical harm to children (Homola 2016) and the usage of herbal compounds causing liver toxicity (Frenzel and Teschke 2016) and nephrotoxicity (Yang et al. 2018). But let’s suppose that we are considering an alternative therapy that has no side effects whatsoever. There are, however, costs to be paid: salaries of alternative practitioners, the working quarters, material (for example, “homeopathic” water can be more expensive than antibiotics) and most of all, time. The time lost for patients dwelling on their alternative therapies might cost them diagnostic delay, disease progression or personal and family time. While the latter is a personal option (providing the patient is duly informed), the two former concern the patient and society: increased healthcare expenditure and resource allocation, and even public health concerns (faux vaccines, untreated contagious diseases, healthcare accessibility). In fact, labour loss due to diagnostic delay, disease progression and lost work days in ineffective treatments have a deep personal and social impact that is sometimes hard to measure directly. The complementary treatment argument has direct and indirect costs that branch into more than healthcare and extend to the workplace and the family dynamics of the patient, leaving very little ethical justification other than the self-justification of the existence of alternative therapy practitioners.

The concept of patients as consumers has been intertwined with patient-centered care. (Gusmano et al. 2019) Referring to doctors as providers, patients as consumers and medicine as a commodity is a fallacy. This fallacy is indirectly used as an argument by alternative therapies advocates, claiming patient choice and of course, placing alternative therapists in the role of the doctor as a commodity provider. However, patients are not in a position shop or research like in a free-market: they are vulnerable, dependent, and the medical science is vast and deeply technical, you need years of study and training to grasp it. The disastrous effects of replacing medical ethics and professionalism with marketing jargon and business ethics are noticeable in public health outcomes in countries with profit-driven healthcare systems (Lim et al. 2018). Furthermore, the quality and results of alternative therapies pale when compared with medicine: the commodities are not the same, starting in research and development investment, medical and scientific knowledge, innovation and progress, to short and long term clinical results. The “providers” are not the same, starting from pre and post-graduate training intensity and quality, scientific knowledge and clinical experience: failure to understand this important difference could be the result of the Dunning–Kruger effect (Kruger and Dunning 1999). Hence, the consumer patient argument in not valid and is ethically challenged. It is ironic that alternative therapies advocates use a free market or patient-centered care as an argument, when the result of such policy may be opposed to the patient’s best interest.

Some qualified and licensed medical doctors also use alternative therapies: but are they ethically allowed to do so? This situation is more delicate because it covers this practice with a glow of scientific validation that does not exist. In fact, the deontology code of the Portuguese medical association (Ordem dos Médicos) is clear (Código Deontológico, Diário da República 2016): article 7 points that decisions should be dictated by science and conscience, while unnecessary treatments should be avoided, article 8 demands *leges artis* concerning scientific preparation and article 10 is explicit stating that doctors should abstain from any ‘acts which are not in accordance with *leges artis*, unless promising data and no alternative exists and patient/legal representative consent was obtained. Article 56 claims that false advertising is forbidden. Alternative therapies are not part of the *leges artis*, have no scientific basis, multiple unbiased studies show lack of efficacy and demonstrate that they are unnecessary and potentially harmful. Publicising alternative therapies as effective is indeed false advertising.

The so called post-truth, as first coined by Steve Tesich (Tesich 1992), is the trendy Orwellian speak of the twenty-first century. In this environment that censors truth to favour political bias, science and scientific thinking are welcomed with a sneering smile. Non-evidence based therapies thrive in this setting if they have the correct politico-economic and social support, while medicine and evidence-based practices [even long-established ones like vaccines (Phadke et al. 2016)] may encounter opposition by misinformed decision-makers and the general population.

## Conclusions

Our future as a species is dependent on how we face the truth using the proper tools (science) and how we apply them to the best of our knowledge toward the common good. If due to social pressure and obscure interests, the barriers between science and quackery are demolished, truth loses meaning and progress is seriously impaired. Especially concerning medical knowledge and medical practice, non-evidence based therapies may harm those in need with a grim conviction validated by deluded peers, instead of helping patients with effective therapies validated by the scientific method.
